# High‐Titer Antinuclear Antibody Defines a Distinct IgG4‐Related Disease Subset With Predilection for Lung and Kidney Involvement

**DOI:** 10.1155/jimr/5243624

**Published:** 2026-10-30

**Authors:** Jie Zhang, Yurong Zhao, Yiwen Wang, Chao Xue, Lidong Hu, Jianglin Zhang, Jian Zhu

**Affiliations:** ^1^ Department of Rheumatology and Immunology The First Medical Center of Chinese PLA General Hospital 28 Fuxing Road, Haidian District Beijing 100853 China 301hospital.com.cn

**Keywords:** antinuclear antibody, clinical phenotype, hypocomplementemia, IgG4-related disease, kidney involvement, lung involvement

## Abstract

**Objective:**

This study investigated whether antinuclear antibody (ANA) positivity identifies a distinct clinical phenotype in IgG4‐related disease (IgG4‐RD), characterized by specific organ involvement patterns.

**Methods:**

We conducted a cohort study of patients diagnosed with IgG4‐RD (2016–2024). Based on indirect immunofluorescence (IIF) results, patients were categorized as ANA‐negative (titer ≤ 1:100) or ANA‐positive, with the latter further stratified by titer: group 1 (moderate titer, 1:320) and group 2 (high titer, ≥1:1000). We comprehensively compared demographics, organ involvement, and laboratory profiles across groups. Multivariable logistic regression was used to quantify associations.

**Results:**

Among 278 patients, 38 (13.7%) were ANA‐positive (*n* = 19 per titer‐based subgroup). ANA patterns differed: granular in group 1 versus homogeneous/granular homogeneous in group 2. Only patients in group 2 (high‐titer ANA) demonstrated a significantly elevated risk of lung and kidney initial onset and involvement compared to ANA‐negative patients. This group also exhibited marked hypocomplementemia. The adjusted odds ratios for lung and kidney involvement in group 2 were 10.18 (95% CI: 3.590–28.876) and 4.42 (95% CI: 1.605–12.151), respectively. No such associations were found in group 1.

**Conclusion:**

IgG4‐RD combined with high‐titer ANA (≥1:1000), mainly presenting as homogeneous type or homogeneous granular type, characterized by a heightened propensity for lung and kidney, initial onset and involvement, and hypocomplementemia. Routine ANA testing and careful assessment of pulmonary and renal systems are warranted in these patients.

## 1. Introduction

IgG4‐related disease (IgG4‐RD) is an immune‐mediated fibroinflammatory disorder characterized by organ enlargement or mass formation, distinctive histopathological features, and elevated serum IgG4 levels [1]. Over the past two decades, research and understanding of IgG4‐RD have progressively advanced. This disease was first formally described by Kamisawa and colleagues in 2003 [2]. In 2014, the journal *Autoimmunity Reviews* published a seminal article titled “The Clinical Spectrum of IgG4‐Related Diseases,” marking its international recognition as a distinct clinical entity [3]. From a pathogenic perspective, IgG4‐RD has an autoimmune basis, with IgG4^+^ B cells and various T cell subsets, particularly CD4^+^ T cells and T follicular helper (Tfh) cells, playing pivotal roles in its development [2]. The disease exhibits a predilection for middle‐aged and elderly males, with a male‐to‐female ratio of ~2:1 [4]. However, the etiology remains incompletely understood, with genetic predisposition, microbial infections, and dysregulated immune responses proposed as potential contributors [5].

Clinically, IgG4‐RD manifests as a heterogeneous spectrum of symptoms, depending on the organs involved. Common target sites include the pancreas, bile ducts, salivary glands, lymph nodes, and retroperitoneum. Pathognomonic features include lymphoplasmacytic infiltration, storiform fibrosis, and obliterative phlebitis [6, 7]. Despite advances in diagnosis, the clinical heterogeneity of IgG4‐RD often leads to delayed or misdiagnosis, underscoring the need for biomarkers to stratify patient subgroups [6]. Antinuclear antibodies (ANA) are a hallmark of systemic autoimmune diseases (SADs). The presence of ANA antibodies often indicates diffuse connective tissue diseases, such as systemic lupus erythematosus (SLE), Sjögren’s syndrome, inflammatory myopathy, systemic sclerosis, and rheumatoid arthritis (RA). The clinical significance of this antibody in patients with IgG4‐RDs remains unclear. While ANA positivity is less common in IgG4‐RD compared to SADs, recent studies have highlighted its potential clinical significance. For instance, a retrospective cohort study found that 14.8% of IgG4‐RD patients had a family history of autoimmune diseases (AID), and these patients exhibited earlier onset and higher ANA positivity rates [8]. However, the clinical significance of ANA positivity in IgG4‐RD has been rarely studied.

Given the immunopathological overlaps between IgG4‐RD and other autoimmune diseases, particularly SLE, the differential diagnosis becomes significantly more challenging when patients test positive for ANA. Therefore, it is necessary for us to conduct research on this group of people to explore the impact of it on the clinical symptom spectrum of the patients. We hypothesized that ANA titers might also stratify clinically meaningful subgroups in IgG4‐RD. To test this hypothesis, we conducted a cohort study of 278 patients diagnosed with IgG4‐RD between January 2016 and June 2024. Demographic characteristics, organ involvement patterns, and laboratory profiles were comprehensively compared across ANA‐titer–stratified groups to investigate whether ANA titer levels are associated with distinct clinical phenotypes in IgG4‐RD.

## 2. Methods

### 2.1. Study Design and Patients

This was a retrospective cohort study conducted at the First Medical Center of Chinese PLA General Hospital. These screened patients were diagnosed with IgG4‐RD in the First Medical Center of Chinese PLA General Hospital between January 2016 and June 2024. All patients were evaluated by experienced rheumatologists. Inclusion criteria were: (1) complete clinical data; (2) fulfillment of the 2019 American College of Rheumatology (ACR)/European League against Rheumatism (EULAR) classification criteria forIgG4‐RD; and (3) availability of ANA testing results from our hospital. Exclusion criteria were: (1) incomplete clinical data, medical history, or laboratory examination records; (2) absence of autoantibody testing performed at our hospital; (3) concomitant diagnosis of SLE, Sjögren’s syndrome, ANCA‐associated vasculitis, or other connective tissue diseases that could explain the clinical manifestations; and (4) presence of disease‐specific autoantibodies (including anti‐dsDNA, anti‐Sm, anti‐SSA/SSB, anti‐RNP, antisynthetase antibodies, anti‐topoisomerase III, anti‐phospholipase A2 receptor, and ANCA) suggestive of alternative diagnoses, unless clinical judgment determined that these antibodies were not associated with a concurrent connective tissue disease. Tissue biopsy was performed in a subset of patients for diagnostic confirmation and to exclude alternative diagnoses, including other connective tissue diseases. Biopsy specimens were reviewed by experienced pathologists and evaluated for the characteristic histopathological features of IgG4‐RD: dense lymphoplasmacytic infiltration, storiform fibrosis, and obliterative phlebitis, with immunohistochemical staining for IgG4^+^ plasma cells (IgG4^+^ cells > 10/high‐power field and IgG4/IgG ratio > 40%). Ethical approval was obtained from the Chinese PLA General Hospital (S2016‐060‐01), and informed consent was secured from all participants or their legal representatives.

### 2.2. Procedures

ANA was detected in our center using HEp‐2 and rat hepatocyte dual matrix indirect immunofluorescence (IIF) assays. Reagents were selected from Aumon (EUROIMMUN, Germany). The dilution method adopted a serial dilution system (doubling dilution). The titer was defined as the highest dilution ratio at which specific fluorescence was observed compared with the negative serum reaction at the same dilution (Supporting Information 1: Table S1). According to the manufacturer’s instructions, an ANA titer of 1:100 was defined as weakly positive, 1:320 as moderately positive, and ≥1:1000 as strongly positive. ANA fluorescence patterns were classified according to the International Consensus on ANA Patterns (ICAP) issued in September 2015 into nuclear fluorescence pattern, cytoplasmic fluorescence pattern, and mitotic fluorescence pattern. Based on these natural titration cutoffs, a tiered stratification was employed: Patients with an ANA titer ≤ 1:100 constituted the ANA‐negative (control) group. ANA‐positive patients were further subdivided into group 1 (moderate titer, 1:320) and group 2 (high titer, ≥1:1000).

### 2.3. Data Collection and Outcome Definitions

Demographic, clinical, laboratory, and imaging data were systematically extracted from the medical records of enrolled patients. Collected demographic and baseline clinical data included gender, age at disease onset, comorbidities, and initial presenting symptoms. Laboratory indicators included blood routine, urine routine, liver and kidney function, amylase, lipase, immunoglobulin, complement, C‐reactive protein (CRP), erythrocyte sedimentation rate (ESR), ANA, and extractable nuclear antigen (ENA) antibody. Organ involvement was assessed and recorded based on a composite of clinical symptoms, characteristic imaging findings, and/or histopathological evidence. Specifically, IgG4‐related lung disease (IgG4‐RLD) was diagnosed when patients met the 2019 ACR/EULAR classification criteria for IgG4‐RD and exhibited characteristic imaging features, including thickening around and between bronchial vessels and chest paravertebral banded soft tissue on computed tomography. Other pulmonary manifestations (e.g., nodules/masses, interstitial pneumonia/fibrosis, and pleural effusion/thickening) were also recorded according to the IgG4‐RD Responder Index [1] but were not considered pathognomonic for IgG4‐RLD.

### 2.4. Statistical Analysis

Statistical analyses were performed using the SPSS software (version 24.0). Continuous variables are presented as mean ± standard deviation or median (interquartile range) based on their distribution and were compared using Student’s *t*‐test or Mann–Whitney *U* test as appropriate. Categorical variables are presented as frequencies (percentages) and compared using the Chi‐square test or Fisher’s exact test. All statistical tests were two‐tailed, and a *p*‐value < 0.05 was considered statistically significant.

## 3. Results

A total of 278 patients were included in the study after screening, including 240 patients in the ANA‐negative group (ANA ≤ 1:100) and 38 patients in the ANA‐positive group (19 patients in total with a titer of 1:320, named group 1; a total of 19 patients with ≥1:1000 were named group 2). ANA karyotype analysis of 38 patients showed (Figure 1 and Supporting Information 1: Table S2) that ANA was dominated by granular type and granular homogeneous type, accounting for 34.2% and 31.6%. Granular type was more common in group 1 (8/19, accounting for 42.1%), and granular homogenous type was more common in group 2 (9/19, accounting for 47.4%).

**Figure 1:**
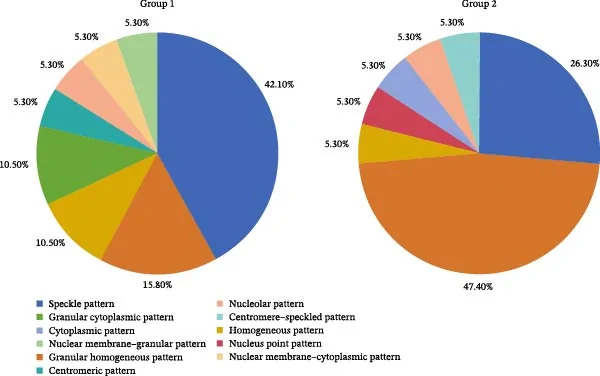
Distribution of antinuclear antibody (ANA) immunofluorescence patterns in IgG4‐related disease (IgG4‐RD) patients stratified by ANA titer. Group 1 (low‐titer, 1:320, *n* = 19) and Group 2 (high‐titer, ≥1:1000, *n* = 19). Values are expressed as percentages within each group. The granular homogeneous pattern was the most frequent in group 2 (47.4%), while the speckled pattern predominated in group 1 (42.1%). Other patterns included homogeneous, nucleolar, centromeric, and various cytoplasmic/nuclear membrane patterns, each occurring at low frequencies.

All patients fulfilled the 2019 ACR/EULAR classification criteria. Specific connective tissue disease antibodies (e.g., anti‐dsDNA, Sm, and SSA/SSB) were systematically assessed. In this study, two patients were observed to have anticentromere antibodies and antinucleosome antibodies and, therefore, were ruled out by the clinician.

### 3.1. Demographic Characteristics

The demographic characteristics of patients in the ANA‐negative and positive groups were compared, and the results showed no statistical differences in sex ratio distribution, age of onset, and comorbidities between the two groups. Subgroup analysis showed a higher proportion of women in group 1 compared with patients in the ANA‐negative group (*p* = 0.031) (Table 1).

**Table 1:** Comparison of demographic characteristics.

Variables	Negative group	Positive group		Group 1		Group 2	
		Value	*p*	Value	*p*	Value	*p*
Male: female	160:80	20:18	0.092	8:11	0.031	12:7	0.755
Age of onset (years)	56.1 ± 11.8	57.8 ± 15.1	0.441	55.1 ± 14.1	0.727	60.5 ± 16.1	0.136
Allergic disease, *n* (%)	75 (31.3)	8 (21.1)	0.202	6 (31.6)	0.976	2 (10.5)	0.057
TB history or T‐SPOT positive, *n* (%)	48 (18.8)	8 (21.1)	0.737	3 (15.8)	1.000	5 (26.3)	0.555
Viral hepatitis B, *n* (%)	8 (3.3)	1 (2.6)	1.000	1 (5.3)	0.502	0	1.000
Cancer, *n* (%)	6 (2.5)	2 (5.3)	0.300	2 (10.5)	0.110	0	1.000

### 3.2. Initial Symptoms

Pancreatic involvement was the most common first symptom in both ANA‐negative and positive groups, followed by salivary gland, ocular, and retroperitoneal fibrosis. The proportion of only pulmonary lesions as the first symptom was significantly higher in the ANA‐positive group than in the ANA‐negative group (7.9% vs. 1.0%, *p* = 0.019). Further analysis showed that this difference was mainly due to group 2 (15.8% vs. 1.0%, *p* = 0.003). In addition, the proportion of patients with kidney disease as the first symptom in group 2 was also higher than that in group negative (15.8% vs. 3.75%, *p* = 0.048) (Table 2).

**Table 2:** Comparison of initial symptoms, specific manifestations, and nonspecific manifestations.

Part/organ	Negative group, *n* (%)	Positive group		Group 1		Group 2	
		Value, *n* (%)	*p*	Value, *n* (%)	*p*	Value, *n* (%)	*p*
Initial symptoms							
Pancreas	102 (42.5)	13 (34.2)	0.335	7 (36.8)	0.631	6 (31.6)	0.353
Salivary gland	45 (18.8)	8 (21.1)	0.737	4 (21.1)	0.765	4 (21.1)	0.765
Eyes	29 (12.1)	6 (15.8)	0.597	3 (15.8)	0.714	3 (15.8)	0.714
Retroperitoneal mass	24 (10.0)	4 (10.5)	1.000	3 (15.8)	0.43	1 (5.3)	1.000
Bile ducts	18 (7.5)	1 (2.6)	0.487	0	0.376	1 (5.3)	1.000
Kidneys	9 (3.75)	3 (7.9)	0.216	0	1.000	3 (15.8)	0.048
Lung	2 (1.0)	3 (7.9)	0.019	0	1.000	3 (15.8)	0.003
Sinuses	2 (1.0)	0	1.000	0	1.000	0	1.000
Pituitary	3 (1.25)	0	1.000	0	1.000	0	1.000
Meninges	2 (1.0)	0	1.000	0	1.000	0	1.000
Lymph node	6 (2.5)	2 (5.3)	0.300	2 (10.5)	0.110	0	1.000
Thyroid	0	1 (7.9)	0.137	1 (5.3)	0.073	0	1.000
Specific manifestations							
Lacrimal/salivary glands	153 (63.8)	22 (57.9)	0.487	10 (52.6)	0.334	12 (63.2)	0.959
One group	55 (22.9)	5 (13.2)	0.174	4 (21.1)	1.000	1 (5.3)	0.085
Two or more groups	98 (40.8)	17 (44.7)	0.650	6 (31.6)	0.428	11 (57.9)	0.147
Orbit or lacrimal gland	65 (27.1)	13 (34.2)	0.364	7 (36.8)	0.361	6 (31.6)	0.672
Salivary glands	135 (56.3)	20 (52.6)	0.676	9 (47.4)	0.453	11 (57.9)	0.889
Chest	16 (6.7)	10 (26.3)	<0.001	2 (10.5)	0.629	8 (42.1)	<0.001
Thickening of perivascular and septal bronchial vessels	7 (2.9)	5 (13.2)	0.014	1 (5.3)	0.461	4 (21.1)	0.005
Chest paravertebral banded soft tissue	9 (3.8)	5 (13.2)	0.029	1 (5.3)	0.540	4 (21.1)	0.010
Pancreas and biliary tract	127 (52.9)	19 (50.0)	0.738	11 (57.9)	0.675	8 (42.1)	0.475
Diffuse swelling of the pancreas	73 (30.4)	13 (34.3)	0.638	6 (31.6)	0.916	7 (36.8)	0.56
Diffuse swollen pancreas with a capsule‐like margin	1 (0.4)	1 (2.6)	0.255	1 (5.3)	0.142	0	1.000
Both pancreas and biliary tree are involved	52 (21.7)	5 (13.2)	0.227	4 (21.1)	1.000	1 (5.3)	0.136
Kidneys	28 (11.7)	9 (23.7)	0.043	2 (10.5)	1.000	7 (36.8)	0.007
Hypocomplementemia	10 (4.2)	5 (13.2)	0.043	0	1.000	5 (26.3)	0.002
Thickening or soft tissue of the renal pelvis	13 (5.4)	2 (5.3)	1.000	1 (5.3)	1.000	1 (5.3)	1.000
Bilateral renal cortical low‐density foci	14 (5.8)	2 (5.3)	1.000	1 (5.3)	1.000	1 (5.3)	1.000
Retroperitoneal	37 (15.4)	4 (10.5)	0.430	2 (10.5)	0.747	2 (10.5)	0.747
Diffuse thickening of the abdominal aortic wall	3 (1.3)	1 (2.6)	0.446	1 (5.3)	0.264	0	1.000
Soft tissue around the abdominal/iliac arteries	34 (14.2)	3 (7.9)	0.290	1 (5.3)	0.485	2 (10.5)	1.000
Nonspecific manifestations							
Dura mater	3 (1.25)	1 (2.6)	0.446	0	1.000	1 (5.3)	0.264
Pituitary	8 (3.33)	1 (2.6)	1.000	0	1.000	1 (5.3)	0.502
Thyroid	9 (3.75)	4 (10.5)	0.085	4 (21.1)	0.010	0	1.000
Lymph nodes	172 (71.7)	26 (68.4)	0.681	11 (57.9)	0.204	15 (78.9)	0.495
Lung^a^	131 (54.6)	24 (63.2)	0.493	9 (47.4)	0.544	15 (78.9)	0.039
Kidney^b^	34 (14.2)	9 (23.7)	0.132	2 (10.5)	1.000	7 (36.8)	0.017
Liver and bile ducts	59 (24.6)	7 (18.4)	0.407	5 (26.3)	0.789	2 (10.5)	0.260

^a^Assessed by the IgG4‐RD Responder Index criteria lung involvements including pulmonary nodules or masses, interstitial pneumonia, pulmonary fibrosis, and pleural effusion/thickening.

^b^Assessed by the IgG4‐RD Responder Index criteria kidney involvements including aseptic pyuria, hematuria, proteinuria (protein quantity not meeting the criteria for nephrotic syndrome), and mass lesions.

### 3.3. Characteristic Clinical Manifestations

Compared with the negative group, the positive group had a higher proportion of the two lung characteristics, including thickening of the peribronchial vessels and septum and hyperplasia of the thoracic paravertebral band soft tissue. When group 1 and group 2 were respectively compared with the negative group, the results showed that only patients in group 2 were different from those in the negative group, with an OR value of 10.182 (95% CI: 3.590–28.876, *p* < 0.001). In addition, the proportion of kidney involvement in the positive group was also higher than that in the negative group, but there was a statistical difference mainly in the proportion of hypocomplementemia. Similarly, after group comparison, only group 2 had a higher proportion of renal involvement (OR = 4.417, 95% CI: 1.605–12.151, *p* = 0.004), the proportion of hypocomplementemia was statistically different from that in the negative group (26.3% vs. 4.2%, *p* = 0.002), while the proportion of renal pelvis and renal cortical placeholder was similar to that in the negative group (Table 2).

### 3.4. Comparison of Clinical Spectrum

There was no statistical difference between the negative group and the positive group in the proportion of each clinical symptom (Table 2). The results showed that the proportion of thyroid lesions in group 1 was higher than that in the negative group (21.1% vs. 3.75%, *p* = 0.010). The incidence of lung involvement and kidney involvement in group 2 was significantly increased (78.9% vs. 54.6%, *p* = 0.039; 36.8% vs. 14.2%, *p* = 0.017).

### 3.5. Laboratory Indicators

Comparative analysis of laboratory indicators before hormone therapy or 6 months after hormone withdrawal between the two groups showed that platelet count and complement C3 and C4 levels in the ANA‐positive group were significantly lower than those in the negative group, and IgG levels were higher than those in the negative group. Moreover, comparison among groups showed that the proportion of lymphocytes in group 1 was higher than that in the negative group, the platelet level was lower than that in the negative group, and there was no statistical difference between the other indexes and the negative group. The level of IgE and complement C3 and C4 in group 2 were significantly lower than those in the negative group, and IgG was higher than that in the negative group. Importantly, all 38 ANA‐positive patients were negative for disease‐specific autoantibodies (anti‐dsDNA, anti‐Sm, anti‐SSA/SSB, anti‐RNP, antisynthetase, antitopoisomerase III, anti‐PLA2R, and ANCA), and none met the classification criteria for SLE, Sjögren’s disease, or ANCA‐associated vasculitis after comprehensive rheumatologic evaluation (Table 3).

**Table 3:** Comparison of laboratory indicators.

Laboratory indicators	Negative group	Positive group		Group 1		Group 2	
		Numerical value	*p*	Numerical value	*p*	Numerical value	*p*
IgG4 (mg/dL)	1110 (470, 1805)	1049 (615, 1675)	0.820	759 (456, 1220)	0.258	1260 (735, 1720)	0.425
White blood cells (10^9^/L)	6.31 (5.17, 7.61)	6.11 ± 2.23	0.140	6.41 ± 2.18	0.494	5.8 ± 2.3	0.141
Lymphocyte ratio	29.1 ± 9.7	30.5 ± 11.0	0.496	37.1 ± 10.0	0.018	27.1 ± 10.2	0.419
Eosinophilic ratio	4.2 (1.9, 8.5)	3.5 (1.7, 6.7)	0.335	3.8 (1.6, 5.4)	0.488	3.1 (1.5, 8.2)	0.470
Neutrophil ratio	56.6 ± 12.1	56.4 ± 12.2	0.938	50.8 ± 8.5	0.164	59.3 ± 13.0	0.385
Hemoglobin (g/L)	131 (114, 140)	121 ± 20	0.090	122 ± 22	0.355	120 ± 20	0.133
Platelets (10^9^/L)	228 ± 50	185 ± 71	0.006	164 ± 83	0.024	197 ± 63	0.059
ALT (U/L)	22 (14, 69)	21 (13, 43)	0.376	40 ± 31	0.852	15 (12, 28)	0.160
AST (U/L)	21 (16, 44)	24 (16, 60)	0.508	36 ± 21	0.326	22 (16, 51)	0.966
AKP (U/L)	83 (61, 196)	86 (59, 165)	0.701	167 ± 123	0.624	81 (55, 112)	0.326
GGT (U/L)	35 (16, 211)	28 (15, 136)	0.342	56 (16, 140)	0.685	18 (11, 143)	0.089
TB (µmol/L)	4 (8, 31)	10 (7, 40)	0.540	13 (7, 124)	0.499	10 (6, 17)	0.143
DB (µmol/L)	4 (2, 20)	4 (2, 30)	0.840	7 (2, 111)	0.403	3 (2, 7)	0.300
BUN (mmol/L)	4.9 (3.8, 6.3)	5.4 (4.1, 6.6)	0.301	4.8 ± 1.3	0.846	5.5 (4.3, 9.3)	0.157
Cr (µmol/L)	70 (60, 84)	74 (60, 92)	0.367	65 (54, 88)	0.459	77 (69, 100)	0.059
Amylase (U/L)	55 (36, 86)	60 (39, 116)	0.499	48 ± 34	0.128	80 (53, 130)	0.058
Lipase (U/L)	77 (33, 202)	54 (33, 180)	0.752	108 ± 109	0.560	54 (35, 157)	1.000
C3 (mg/dL)	100 (83, 121)	83 (53, 118)	0.011	102 ± 32	0.946	67 ± 40	<0.001
C4 (mg/dL)	20 (13, 27)	13 (4, 18)	<0.001	17 ± 10	0.152	8 (2, 16)	<0.001
IgG (mg/dL)	1810 (1410, 2435)	2005 (1710, 3035)	0.021	1890 (1527, 2485)	0.741	2530 (1935, 3550)	0.002
IgE (mg/dL)	197 (81, 616)	123 (53, 387)	0.095	182 (79, 624)	0.571	105 (41, 283)	0.042
CRP (mg/dL)	0.32 (0.15, 0.57)	0.32 (0.15, 0.42)	0.783	0.32 (0.10, 0.47)	0.682	0.32 (0.30, 0.54)	0.415
ESR (mm/h)	11 (10, 25)	11 (10, 21)	0.760	22 ± 19	0.430	10 (9, 13)	0.329

*Note:* Mean ± SD/median (IQR).

### 3.6. Lung and Kidney Involvement

In the previous statistical results, we found that there were significant differences in lung and kidney involvement between group 2 and the negative group, so we further conducted subgroup analysis of these two systems. In terms of lung involvement, pulmonary nodules/masses and interstitial pneumonia/fibrosis were common manifestations in both groups, and there was no statistical difference between the two groups. The two characteristic changes of bronchial vascular bundle changes and thoracic paravertebral soft tissue shadow were more common in group 2 than in negative group (26.7% vs. 5.3%, *p* = 0.016; 26.7% vs. 6.9, *p* = 0.030). Representative images for an IgG4‐RD patient with multisystem involvement are provided in Supporting Information 2: Figure S1.

In terms of renal performance, the proportion of small amounts of proteinuria in group 2 was significantly higher than in the negative group (57.1% vs. 5.9%, *p* = 0.005), the proportion and extent of creatinine elevation was not statistically different between the two groups, and although the proportion of complement decline was comparable between the two groups, the complement C3 and C4 levels in group 2 were lower than those in the negative group (Table 4).

**Table 4:** Comparison of lung and kidney involvement.

Clinical picture	Negative group	Group 2	
		Value	*p*
Lung manifestations	***n* = 131**	***n* = 15**	
Thickening around and between bronchial vessels, *n* (%)	7 (5.3)	4 (26.7)	0.016
Chest paravertebral band soft tissue, *n* (%)	9 (6.9)	4 (26.7)	0.030
Lung nodule or mass, *n* (%)	74 (56.5)	8 (53.3)	0.816
Interstitial pneumonia/fibrosis^a^, *n* (%)	48 (36.6)	7 (46.7)	0.448
Pleural effusion or pleural thickening, *n* (%)	24 (18.3)	2 (13.3)	1.000
Kidney	***n* = 34**	***n* = 7**	
Thickened pelvis or soft tissue (mass lesions), *n* (%)	13 (38.2)	1 (14.3)	0.389
Bilateral renal cortical low‐density foci, *n* (%)	14 (41.2)	1 (14.3)	0.232
Other renal imaging abnormalities^b^, *n* (%)	11 (32.4)	0	0.160
Sterile pyuria, *n* (%)	9 (26.5)	3 (42.9)	0.398
Hematuria, *n* (%)	11 (32.4)	2 (28.6)	1.000
Proteinuria (not meeting criteria for nephrotic syndrome), *n* (%)	2 (5.9)	4 (57.1)	0.005
Large proteinuria (meeting criteria for nephrotic syndrome), *n* (%)	3 (8.8)	0	1.000
Creatinine increases in proportion, *n* (%)	16 (47.1)	2 (28.6)	0.438
Creatinine level (μmol/L), median (IQR)	85.7 (71.8, 134.1)	83.4 (62.5, 229.8)	0.918
Decreased proportion of complement C3, *n* (%)	22 (64.7)	6 (85.7)	0.399
Complement C3 levels (mg/dL), median (IQR)	94.1 (69.2, 116.3)	34.9 (30.5, 87.0)	0.007
Proportion of complement C4 decreased, *n* (%)	12 (35.3)	5 (71.4)	0.105
Complement C4 level (mg/dL), median (IQR)	19 (8.5, 25.3)	2.54 (1.7, 15.2)	0.016

*Note:* The bold values represent the number of patients with pulmonary and renal involvement in the negative group and group 2, respectively.

^a^Interstitial pneumonia/fibrosis: interstitial changes in addition to changes around bronchial vascular bundles, including bronchiolitis, ground glass shadow, downline pleura, honeycomb changes, and thickening of interlobular septa.

^b^Other renal imaging abnormalities: ultrasound, MRI, or PET‐CT examination suggests abnormal renal morphology, abnormal size, abnormal signaling or abnormal uptake, perirenal fascia thickening, and perirenal exudation.

### 3.7. Histopathological Findings

Among the 278 patients, tissue biopsy was performed in 186 patients (66.9%), involving the following organs: pancreas (*n* = 68), salivary glands (*n* = 45), lymph nodes (*n* = 28), kidney (*n* = 15), lung (*n* = 12), lacrimal glands (*n* = 10), retroperitoneal tissue (*n* = 5), bile duct (*n* = 3), and others (*n* = 5). Importantly, no biopsy specimen showed histopathological evidence of lupus nephritis, ANCA‐associated glomerulonephritis, lymphoproliferative disorders, or other connective tissue diseases. In the ANA‐positive group, 26 of 38 patients (68.4%) underwent biopsy, and all confirmed IgG4‐RD without evidence of alternative diagnoses.

## 4. Discussion

This study revealed that high‐titer ANA (≥1:1000) might be associated with increased risks of lung and kidney involvement, as well as hypocomplementemia. These findings highlight the importance of ANA titers as a potential biomarker for identifying subgroups of IgG4‐RD patients with distinct organ‐specific manifestations and immune dysregulation, which may guide targeted diagnostic and therapeutic strategies.

In our study, the homogeneous and granular homogeneous ANA patterns predominated in the high‐titer group. Previous literature has indicated that the homogeneous ANA pattern is frequently associated with anti‐dsDNA, antihistone, and antinucleosome antibodies, which are potent activators of the classical complement pathway [8–10]. Notably, all 38 ANA‐positive patients in our cohort were negative for disease‐specific autoantibodies (anti‐dsDNA, anti‐Sm, anti‐SSA/SSB, etc.) and did not meet classification criteria for SLE, Sjögren’s disease, or ANCA‐associated vasculitis, confirming that these findings represent a genuine IgG4‐RD phenotype rather than an overlapping connective tissue disease. The predominance of homogeneous pattern in high‐titer ANA suggests that these antibodies may have enhanced complement‐activation capacity. Consistent with this possibility, we observed that patients in group 2 (high‐titer ANA) had significantly lower C3 and C4 levels compared to ANA‐negative patients. Hypocomplementemia in IgG4‐RD has been linked to multiorgan involvement, particularly affecting the lungs and kidneys [11, 12]. Mechanistically, high‐titer ANA could bind to corresponding antigens, forming immune complexes that deposit in the capillary beds of the lungs and kidneys, organs with rich blood supply, potentially triggering the complement cascade and contributing to local inflammation and tissue injury. Although the main renal lesion in IgG4‐RD is tubulointerstitial nephritis rather than glomerulonephritis as seen in lupus nephritis, the mechanism of complement consumption may share similarities, and both may involve complement activation following immune complex deposition. In addition, type I hypersensitivity has also been proposed to play a role in the pathogenesis of IgG4‐RD [13, 14].

Further supporting this immune complex–complement axis, total IgG levels were significantly elevated in the high‐titer ANA group, yet IgG4 levels did not differ from controls, suggesting a possible shift toward complement‐fixing IgG subclasses. Previous studies have shown that ANA in IgG4‐RD belongs predominantly to the IgG1 and IgG2 subclasses rather than IgG4 [6]. This subclass distribution has functional implications: IgG1 and IgG2 are efficient complement fixers with strong binding affinity for C1q, whereas IgG4 is a poor complement activator. Therefore, high‐titer ANA dominated by IgG1/IgG2 could possess a greater capacity for complement activation, potentially making it more likely to trigger complement‐mediated inflammatory injury in target organs. Moreover, Tfh cells, particularly the Tfh1 subset, are expanded in IgG4‐RD and promote IgG1 and IgG2 production via IFN‐γ [13, 15]. IFN‐γ also upregulates adhesion molecules on endothelial cells, potentially enhancing leukocyte recruitment to the lung and kidney microvasculature. Studies have shown that IFN‐γ produced by Tfh1 further promotes the production of the IgG2 subclass, and IgG2‐positive B cells are observed in tissues [16, 17]. Thus, the Tfh1‐IgG1/IgG2‐complement activation axis may represent a potential immune mechanism through which high‐titer ANA contributes to lung and kidney involvement.

The selective nature of the pulmonary findings provides additional support for this mechanistic hypothesis. Among the various pulmonary imaging findings, only the two characteristic features, thickening around and between bronchial vessels and chest paravertebral banded soft tissue, were significantly associated with high‐titer ANA, while other pulmonary manifestations such as nodules/masses, interstitial pneumonia/fibrosis, and pleural effusion/thickening did not differ between groups. Previous literatures have indicated that bronchovascular bundle thickening and paravertebral soft tissue are characteristic pulmonary lesions of IgG4‐RD, with a pathological basis of lymphoplasmacytic infiltration and fibrosis distributed along the bronchovascular bundles and subpleural spaces [18, 19]. The rich blood supply in these areas may make them susceptible sites for circulating immune complex deposition and complement‐mediated inflammation. In contrast, nodules, fibrosis, and pleural thickening are nonspecific findings commonly observed in other conditions, and their pathogenesis may be unrelated to the specific immune processes of IgG4‐RD. We recorded these noncharacteristic manifestations according to the parameters of the IgG4‐RD Responder Index [20, 21]. Therefore, the observation that ANA was associated only with characteristic lesions and not with nonspecific findings offers supportive evidence for the hypothesis that high‐titer ANA may selectively affect blood‐rich organs through immune complex–complement pathway activation.

## 5. Limitations

This study has several limitations. First, this study relied on retrospective data, limiting causal inferences about the role of ANA in disease progression, and prospective longitudinal studies are required to validate these associations. Second, the cohort is primarily Chinese, and the results may not generalize to other populations. Third, the absence of Tfh cell counts, cytokine levels, or tissue‐based immune analyses restricts mechanistic insights. Fourth, serum circulating immune complexes were not measured in our center, preventing direct evaluation of complement activation pathways; however, we plan to examine tissue specimens for evidence of immune complex deposition in future studies. Fifth, although ENA positivity was rare, the potential for undiagnosed early‐stage connective tissue diseases cannot be entirely excluded [22]. Sixth, data on clinical features such as family history and thyroid involvement, which may be associated with ANA status, were not included in the analysis.

## 6. Conclusion

In conclusion, this study found that high‐titer ANA (≥1:1000) in IgG4‐RD might be associated with lung and kidney initial onset and involvement, hypocomplementemia, and distinct immunological profiles. These findings suggest that clinicians could consider prioritizing pulmonary and renal evaluations in IgG4‐RD patients with high‐titer ANA to facilitate early detection of organ involvement. Future prospective studies incorporating comprehensive immune profiling and multiomics approaches are needed to further elucidate the underlying pathogenic mechanisms.

## Author Contributions

All authors (Jie Zhang, Yurong Zhao, Yiwen Wang, Chao Xue, Lidong Hu, Jianglin Zhang, and Jian Zhu) contributed to this work. Specifically, all authors participated in the conceptualization, investigation, funding acquisition, writing – original draft, visualization, validation, methodology, writing – review and editing, formal analysis, software, project administration, data curation, supervision, and resources.

## Funding

This work was supported by the National Natural Science Foundation of China (Grant 82302028), the Beijing Natural Science Foundation (7242036), and the Chinese PLA General Hospital Fourth Medical Center Independent Innovation Science Fund (Grant 2024‐4ZX‐MS‐18).

## Disclosure

All authors have read and approved the final manuscript. All content was originally produced by the authors.

## Ethics Statement

Ethical approval was obtained from the Chinese PLA General Hospital (S2016‐060‐01), and informed consent was secured from all participants or their legal representatives.

## Conflicts of Interest

The authors declare no conflicts of interest.

## Supporting Information

Additional supporting information can be found online in the Supporting Information section.

## Supporting information


**Supporting Information 1** Table S1: The fluorescence intensity grading (weak, moderate, and strong) and corresponding ANA titers in a serial dilution system (1:10 to 1:10,000). It serves as a standardized reference for semiquantitative ANA titer determination based on immunofluorescence patterns. Table S2: The distribution of ANA immunofluorescence patterns in IgG4‐RD patients stratified by ANA titer percentiles (top 1% to bottom 100%). It lists the frequencies of specific patterns (e.g., homogeneous, speckled, and nucleolar) per percentile group, enabling assessment of pattern–titer associations and revealing serological heterogeneity in IgG4‐RD.

**Supporting Information 2** Figure S1: Representative CT images from an IgG4‐RD patient with multisystem involvement: (A) chest CT—paravertebral soft tissue proliferation (yellow arrows), (B) abdominal CT—periaortic (yellow arrows) and renal hilar (yellow asterisks) soft tissue proliferation, and (C) chest CT—bronchial wall and peribronchovascular bundle thickening in the right middle lobe (yellow arrows).

## Data Availability

Research data are not shared.
