# Supplementary material for: High‐Titer Antinuclear Antibody Defines a Distinct IgG4‐Related Disease Subset With Predilection for Lung and Kidney Involvement
Source: J Immunol Res. 2026 Oct 30;2026:5243624. doi: 10.1155/jimr/5243624 (PMC13720895; doi:10.1155/jimr/5243624)
Supplement: Supplementary file 1 [file JIMR-2026-5243624-s001.docx]

**Table S1 Fluorescence intensity grading and corresponding ANA titers in the serial dilution system**

| Dilution | | | | Reported titer |
| --- | --- | --- | --- | --- |
| 1:10 | 1:100 | 1:1000 | 1:10000 |  |
| Weak | Negative | Negative | Negative | 1:10 |
| Moderate | Weak | Negative | Negative | 1:100 |
| Strong | Moderate | Negative | Negative | 1:320 |
| Strong | Strong | Weak | Negative | 1:1000 |
| Strong | Strong | Moderate | Negative | 1:3200 |
| Strong | Strong | Strong | Weak | 1:10000 |

**Table S2 Distribution of antinuclear antibody (ANA) immunofluorescence patterns in IgG4-related disease (IgG4-RD) patients stratified by ANA titer**

| **Nuclear pattern n (%)** | **Goup1** | **Goup2** | **Total** |
| --- | --- | --- | --- |
| **Speckle pattern** | 8(42.1) | 5(26.3) | 13(34.2) |
| **Granular Homogeneous pattern** | 3(15.8) | 9(47.4) | 12(31.6) |
| **Homogeneous pattern** | 2(10.5) | 1(5.3) | 3(7.9) |
| **Granular cytoplasmic pattern** | 2(10.5) | 0 | 2(5.3) |
| **Centromeric pattern** | 1(5.3) | 0() | 1(2.6) |
| **Nucleus Point pattern** | 0 | 1(5.3) | 1(2.6) |
| **Cytoplasmic pattern** | 0 | 1(5.3) | 1(2.6) |
| **nucleolar pattern** | 1(5.3) | 1(5.3) | 2(5.3) |
| **Nuclear membrane-cytoplasmic pattern** | 1(5.3) | 0 | 1(2.6) |
| **Nuclear membrane–granular pattern** | 1(5.3) | 0 | 1(2.6) |
| **Centromere-speckled pattern** | 0 | 1(5.3) | 1(2.6) |
