# Supplementary material for: High‐Titer Antinuclear Antibody Defines a Distinct IgG4‐Related Disease Subset With Predilection for Lung and Kidney Involvement
Source: J Immunol Res. 2026 Oct 30;2026:5243624. doi: 10.1155/jimr/5243624 (PMC13720895; doi:10.1155/jimr/5243624)
Supplement: Supplementary file 2 [file JIMR-2026-5243624-s002.docx]

**Fig S1. Representative images in a IgG4-RD patient with multisystem involvement.** A. Chest CT showing paravertebral soft tissue proliferation (yellow arrows). B. Abdominal CT revealing periaortic soft tissue proliferation (yellow arrows) and renal hilar soft tissue proliferation (yellow asterisks). C. Chest CT demonstrating bronchial wall thickening and peribronchovascular bundle thickening in the right middle lobe (yellow arrows)

A

B

C
